# 2-Amino-5-fluoro­benzoic acid

**DOI:** 10.1107/S160053681300408X

**Published:** 2013-02-16

**Authors:** S. Sreenivasa, K.E. ManojKumar, P.A. Suchetan, B. S. Palakshamurthy, K. Gunasekaran

**Affiliations:** a Department of Studies and Research in Chemistry, Tumkur University, Tumkur, Karnataka 572 103, India; b Department of Studies and Research in Chemistry, U.C.S., Tumkur University, Tumkur, Karnataka 572 103, India; c Department of Studies and Research in Physics, U.C.S., Tumkur University, Tumkur, Karnataka 572 103, India; d Centre of Advanced Study in Crystallography and Biophysics, University of Madras Guindy Campus, Chennai 600 025, India

## Abstract

In the title compound, C_7_H_6_FNO_2_, the mol­ecule is almost planar (r.m.s. deviation for the non-H atoms = 0.015 Å) and an intra­molecular N—H⋯O hydrogen bond closes an *S*(6) ring. In the crystal, inversion dimers linked by pairs of O—H⋯O hydrogen bonds generate *R*_2_^2^(8) loops. Weak N—H⋯F hydrogen bonds, short F⋯F contacts [2.763 (2) Å] and aromatic π–π stacking inter­actions [centroid–centroid separation = 3.5570 (11) Å] are also observed in the crystal structure.

## Related literature

For the applications of the title compound in the field of genetics, see: Toyn *et al.* (2000 ▶).
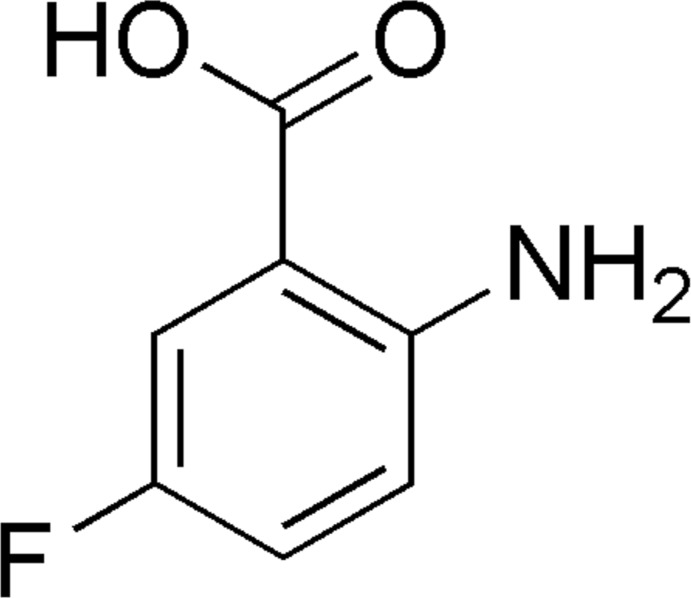


## Experimental

### 

#### Crystal data

C_7_H_6_FNO_2_
*M* *_r_* = 155.13
Monoclinic, 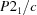

*a* = 4.9346 (2) Å
*b* = 11.7542 (6) Å
*c* = 11.9727 (5) Å
β = 96.782 (3)°
*V* = 689.58 (5) Å^3^
*Z* = 4
Mo *K*α radiation
μ = 0.13 mm^−1^
*T* = 293 K
0.43 × 0.37 × 0.25 mm

#### Data collection

Bruker APEXII CCD diffractometer
Absorption correction: multi-scan (*SADABS*; Bruker, 2004 ▶) *T*_min_ = 0.947, *T*_max_ = 0.969
5184 measured reflections
1207 independent reflections
1057 reflections with *I* > 2σ(*I*)
*R*_int_ = 0.025

#### Refinement

*R*[*F* ^2^ > 2σ(*F* ^2^)] = 0.034
*wR*(*F* ^2^) = 0.100
*S* = 1.09
1207 reflections
108 parameters
H atoms treated by a mixture of independent and constrained refinement
Δρ_max_ = 0.11 e Å^−3^
Δρ_min_ = −0.18 e Å^−3^

### 

Data collection: *APEX2* (Bruker, 2004 ▶);; cell refinement: *SAINT-Plus* (Bruker, 2004 ▶); data reduction: *SAINT-Plus*; program(s) used to solve structure: *SHELXS97* (Sheldrick, 2008 ▶); program(s) used to refine structure: *SHELXL97* (Sheldrick, 2008 ▶); molecular graphics: *ORTEP-3 for Windows* (Farrugia, 2012 ▶); software used to prepare material for publication: *SHELXL97*.

## Supplementary Material

Crystal structure: contains datablock(s) I, global. DOI: 10.1107/S160053681300408X/hb7040sup1.cif

Structure factors: contains datablock(s) I. DOI: 10.1107/S160053681300408X/hb7040Isup2.hkl

Supplementary material file. DOI: 10.1107/S160053681300408X/hb7040Isup3.cml

Additional supplementary materials:  crystallographic information; 3D view; checkCIF report

## Figures and Tables

**Table 1:** Hydrogen-bond geometry (Å, °)

*D*—H⋯*A*	*D*—H	H⋯*A*	*D*⋯*A*	*D*—H⋯*A*
N1—H1*B*⋯O1	0.901 (19)	2.044 (19)	2.6959 (17)	128.2 (16)
N1—H1*A*⋯F1^i^	0.91 (2)	2.55 (2)	3.3646 (17)	149.8 (14)
O2—H2⋯O1^ii^	0.82	1.81	2.6279 (12)	175

Symmetry codes: (i) 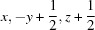; (ii) 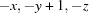.

**Fig. 1.**
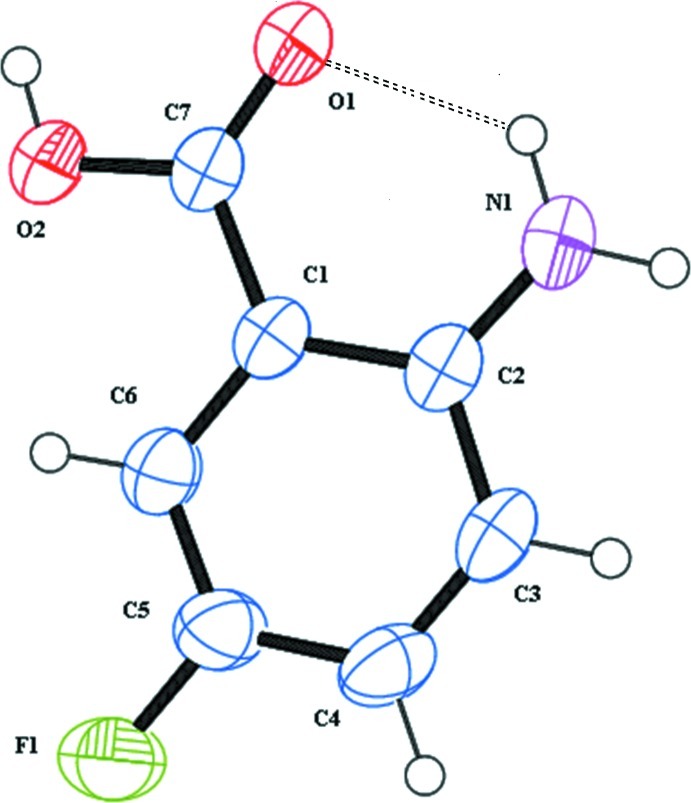
Molecular structure of the title compound, with displacement ellipsoids drawn at the 50% probability level.

**Fig. 2.**
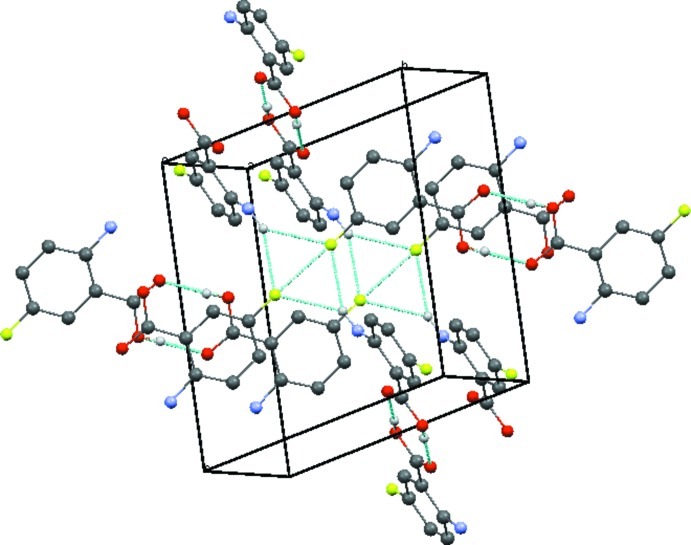
Molecular packing in the title compound. Hydrogen bonds are shown as dashed lines

**Fig. 3.**
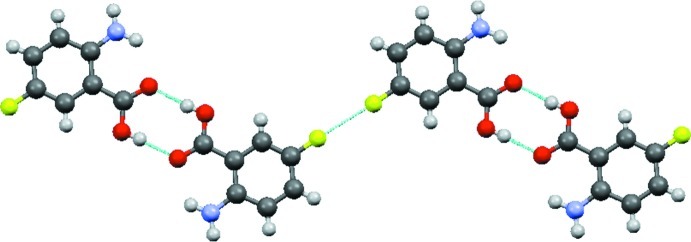
Display of F···F contacts and O–H···O Hydrogen bonds.

## supplementary crystallographic information

### Comment

2-Amino-5-fluorobenzoic acid is used for the counterselection of TRP1, a commonly used genetic marker in the yeast Saccharomyces cerevisiae (Toyn *et al.*, 2000). The ability to counterselect, as well as to select for, a genetic marker has numerous applications in microbial genetics. Keeping this in mind, the structure of the title compound is discussed here.

In the crystal structure of the title compound, C_7_H_6_FNO_2_, the molecules are linked through O2—H2···O1 hydrogen bonds into inversion related dimers. N1—H1A···F1 hydrogen bonds and short F1···F1 contacts [2.763 (2) Å] are also observed in the crystal structure. Further, the structure features an intra molecular hydrogen bond between the amino proton and the adjacent carboxylic oxygen atom.

### Experimental

4-Fluoroaniline (0.01 mmol), aluminium chloride (0.05 mmol) and trichloroacetyl chloride(0.03 mmol) were taken in dichloro methane (DCM) (20 ml) at 00 C under nitrogen atmosphere. The reaction mixture was refluxed for 16 h. The mixture was poured into ice-water carefully and the pH was adjusted to 2. The organic layer was separated and the aqueous layer was extracted with DCM. The combined extract was concentrated to get dark oily 1-(2-amino-5-fluorophenyl)-2,2,2-trichloroethanone. This compound (0.01 mmol) was dissolved in methanol. To this solution sodium methoxide (25% w/t in methanol) was added at 0 °C. The mixture was stirred for 1 h, the pH was adjusted to 6. The resulting solution was extracted with DCM and concentrated to get a brown solid methyl-2-amino-5-fluorobenzoate. To a solution of methyl-2-amino-5-fluorobenzoate (0.01 mmol) in tetrahydrofuran (10 ml),1 N Lithium hydroxide (0.03 mmol) was added at room temperature and stirred at 60 °C for 6 h. The reaction mixture was cooled to 0 °C and pH was adjusted to 5, the precipitate obtained was collected and dried in vacuum to get the title compound. Colourless prisms were obtained by slow evaporation of the solution of the compound in a mixture of DCM and methanol.

### Refinement

The H atoms were positioned with idealized geometry using a riding model with C—H = 0.93 Å. All H atoms were refined with isotropic displacement parameters (set to 1.2 times of the *U*_eq_ of the parent atom).

### Crystal data

**Table:**

C_7_H_6_FNO_2_	Prism
*M**_r_* = 155.13	*D*_x_ = 1.494 Mg m^−^^3^
Monoclinic, *P*2_1_/*c*	Melting point: 454 K
Hall symbol: -P 2ybc	Mo *K*α radiation, λ = 0.71073 Å
*a* = 4.9346 (2) Å	Cell parameters from 108 reflections
*b* = 11.7542 (6) Å	θ = 2.4–25.0°
*c* = 11.9727 (5) Å	µ = 0.13 mm^−^^1^
β = 96.782 (3)°	*T* = 293 K
*V* = 689.58 (5) Å^3^	Prism, colourless
*Z* = 4	0.43 × 0.37 × 0.25 mm
*F*(000) = 320	

### Data collection

**Table:**

Bruker APEXII CCD diffractometer	1207 independent reflections
Radiation source: fine-focus sealed tube	1057 reflections with *I* > 2σ(*I*)
Graphite monochromator	*R*_int_ = 0.025
ω scans	θ_max_ = 25.0°, θ_min_ = 2.4°
Absorption correction: multi-scan (*SADABS*; Bruker, 2004)	*h* = −5→5
*T*_min_ = 0.947, *T*_max_ = 0.969	*k* = −13→13
5184 measured reflections	*l* = −14→14

### Refinement

**Table:**

Refinement on *F*^2^	Primary atom site location: structure-invariant direct methods
Least-squares matrix: full	Secondary atom site location: difference Fourier map
*R*[*F*^2^ > 2σ(*F*^2^)] = 0.034	Hydrogen site location: inferred from neighbouring sites
*wR*(*F*^2^) = 0.100	H atoms treated by a mixture of independent and constrained refinement
*S* = 1.09	*w* = 1/[σ^2^(*F*_o_^2^) + (0.0616*P*)^2^ + 0.0629*P*] where *P* = (*F*_o_^2^ + 2*F*_c_^2^)/3
1207 reflections	(Δ/σ)_max_ < 0.001
108 parameters	Δρ_max_ = 0.11 e Å^−^^3^
0 restraints	Δρ_min_ = −0.18 e Å^−^^3^
0 constraints	

### Special details

**Table:**

Geometry. All e.s.d.'s (except the e.s.d. in the dihedral angle between two l.s. planes) are estimated using the full covariance matrix. The cell e.s.d.'s are taken into account individually in the estimation of e.s.d.'s in distances, angles and torsion angles; correlations between e.s.d.'s in cell parameters are only used when they are defined by crystal symmetry. An approximate (isotropic) treatment of cell e.s.d.'s is used for estimating e.s.d.'s involving l.s. planes.
Refinement. Refinement of *F*^2^ against ALL reflections. The weighted *R*-factor *wR* and goodness of fit *S* are based on *F*^2^, conventional *R*-factors *R* are based on *F*, with *F* set to zero for negative *F*^2^. The threshold expression of *F*^2^ > σ(*F*^2^) is used only for calculating *R*-factors(gt) *etc*. and is not relevant to the choice of reflections for refinement. *R*-factors based on *F*^2^ are statistically about twice as large as those based on *F*, and *R*- factors based on ALL data will be even larger.

### Fractional atomic coordinates and isotropic or equivalent isotropic displacement parameters (Å^2^)

**Table:**

	*x*	*y*	*z*	*U*_iso_*/*U*_eq_	
H1B	0.443 (4)	0.5012 (15)	0.2698 (16)	0.077 (5)*	
H1A	0.681 (4)	0.4717 (14)	0.3630 (16)	0.078 (5)*	
O1	0.20891 (18)	0.49475 (8)	0.11724 (8)	0.0572 (3)	
C1	0.5279 (2)	0.34252 (10)	0.12427 (10)	0.0419 (3)	
O2	0.20142 (19)	0.37877 (9)	−0.02978 (7)	0.0606 (3)	
H2	0.0757	0.4209	−0.0541	0.091*	
C2	0.6596 (2)	0.36817 (11)	0.23280 (10)	0.0458 (3)	
C6	0.6146 (3)	0.24937 (12)	0.06514 (10)	0.0515 (3)	
H6	0.5277	0.2315	−0.0059	0.062*	
N1	0.5831 (3)	0.45571 (12)	0.29601 (11)	0.0688 (4)	
C7	0.3008 (2)	0.41184 (10)	0.07158 (10)	0.0430 (3)	
C4	0.9633 (3)	0.20846 (12)	0.21735 (11)	0.0561 (4)	
H4	1.1092	0.1636	0.2474	0.067*	
C3	0.8792 (3)	0.29852 (12)	0.27596 (11)	0.0529 (4)	
H3	0.9699	0.3145	0.3468	0.063*	
F1	0.9117 (2)	0.09467 (9)	0.05376 (8)	0.0924 (4)	
C5	0.8272 (3)	0.18517 (12)	0.11232 (11)	0.0567 (4)	

### Atomic displacement parameters (Å^2^)

**Table:**

	*U*^11^	*U*^22^	*U*^33^	*U*^12^	*U*^13^	*U*^23^
O1	0.0586 (6)	0.0593 (6)	0.0492 (6)	0.0139 (4)	−0.0129 (4)	−0.0088 (4)
C1	0.0393 (6)	0.0479 (7)	0.0374 (6)	−0.0016 (5)	0.0000 (5)	0.0045 (5)
O2	0.0619 (6)	0.0706 (6)	0.0439 (6)	0.0196 (5)	−0.0163 (4)	−0.0100 (4)
C2	0.0430 (6)	0.0513 (7)	0.0413 (7)	−0.0038 (5)	−0.0023 (5)	0.0027 (5)
C6	0.0566 (7)	0.0593 (7)	0.0370 (6)	0.0071 (6)	−0.0017 (5)	0.0009 (6)
N1	0.0719 (8)	0.0721 (8)	0.0549 (8)	0.0145 (7)	−0.0238 (6)	−0.0188 (6)
C7	0.0407 (6)	0.0477 (7)	0.0387 (6)	−0.0033 (5)	−0.0024 (5)	0.0009 (5)
C4	0.0538 (7)	0.0674 (9)	0.0461 (7)	0.0148 (6)	0.0018 (6)	0.0161 (6)
C3	0.0507 (7)	0.0664 (8)	0.0384 (6)	0.0003 (6)	−0.0077 (5)	0.0079 (6)
F1	0.1194 (8)	0.0939 (7)	0.0597 (6)	0.0593 (6)	−0.0071 (5)	−0.0108 (5)
C5	0.0665 (8)	0.0594 (8)	0.0442 (7)	0.0186 (6)	0.0061 (6)	0.0048 (6)

### Geometric parameters (Å, º)

**Table:**

O1—C7	1.2299 (15)	C6—H6	0.9300
C1—C6	1.3982 (18)	N1—H1B	0.901 (19)
C1—C2	1.4150 (17)	N1—H1A	0.91 (2)
C1—C7	1.4667 (16)	C4—C3	1.361 (2)
O2—C7	1.3129 (14)	C4—C5	1.381 (2)
O2—H2	0.8200	C4—H4	0.9300
C2—N1	1.3571 (19)	C3—H3	0.9300
C2—C3	1.4069 (18)	F1—C5	1.3660 (16)
C6—C5	1.3599 (18)		
			
C6—C1—C2	119.80 (11)	O1—C7—O2	121.90 (10)
C6—C1—C7	118.77 (10)	O1—C7—C1	123.51 (10)
C2—C1—C7	121.43 (11)	O2—C7—C1	114.59 (11)
C7—O2—H2	109.5	C3—C4—C5	118.59 (11)
N1—C2—C3	119.29 (11)	C3—C4—H4	120.7
N1—C2—C1	123.08 (11)	C5—C4—H4	120.7
C3—C2—C1	117.63 (12)	C4—C3—C2	122.10 (11)
C5—C6—C1	119.44 (12)	C4—C3—H3	119.0
C5—C6—H6	120.3	C2—C3—H3	119.0
C1—C6—H6	120.3	C6—C5—F1	119.08 (12)
C2—N1—H1B	120.5 (12)	C6—C5—C4	122.44 (13)
C2—N1—H1A	119.8 (11)	F1—C5—C4	118.48 (12)
H1B—N1—H1A	119.5 (17)		
			
C6—C1—C2—N1	−178.62 (12)	C2—C1—C7—O2	179.10 (11)
C7—C1—C2—N1	1.46 (19)	C5—C4—C3—C2	−0.1 (2)
C6—C1—C2—C3	1.17 (18)	N1—C2—C3—C4	179.06 (13)
C7—C1—C2—C3	−178.75 (11)	C1—C2—C3—C4	−0.74 (19)
C2—C1—C6—C5	−0.75 (19)	C1—C6—C5—F1	−179.65 (12)
C7—C1—C6—C5	179.17 (11)	C1—C6—C5—C4	−0.1 (2)
C6—C1—C7—O1	179.42 (11)	C3—C4—C5—C6	0.6 (2)
C2—C1—C7—O1	−0.66 (18)	C3—C4—C5—F1	−179.91 (12)
C6—C1—C7—O2	−0.82 (16)		

### Hydrogen-bond geometry (Å, º)

**Table:**

*D*—H···*A*	*D*—H	H···*A*	*D*···*A*	*D*—H···*A*
N1—H1*B*···O1	0.901 (19)	2.044 (19)	2.6959 (17)	128.2 (16)
N1—H1*A*···F1^i^	0.91 (2)	2.55 (2)	3.3646 (17)	149.8 (14)
O2—H2···O1^ii^	0.82	1.81	2.6279 (12)	175

Symmetry codes: (i) *x*, −*y*+1/2, *z*+1/2; (ii) −*x*, −*y*+1, −*z*.
